# Pax3 deficiency diminishes melanocytes in the developing mouse cochlea

**DOI:** 10.21203/rs.3.rs-2990436/v1

**Published:** 2023-06-09

**Authors:** Tomokatsu Udagawa, Erisa Takahashi, Norifumi Tatsumi, Hideki Mutai, Yuko Kondo, Patrick J. Atkinson, Tatsuo Matsunaga, Mamoru Yoshikawa, Hiromi Kojima, Masataka Okabe, Alan G. Cheng

## Abstract

Cochlear melanocytes are intermediate cells in the stria vascularis that generate endocochlear potentials required for auditory function. Human PAX3 mutations cause Waardenburg syndrome and abnormalities of melanocytes, manifested as congenital hearing loss and hypopigmentation of skin, hair and eyes. However, the underlying mechanism of hearing loss remains unclear. During development, cochlear melanocytes in the stria vascularis are dually derived from Pax3-Cre+ melanoblasts migrating from neuroepithelial cells including neural crest cells and Plp1+ Schwann cell precursors originated from also neural crest cells, differentiating in a basal-apical manner. Here, using a Pax3-Cre mouse line, we found that Pax3 deficiency causes foreshortened cochlea, malformed vestibular apparatus, and neural tube defects. Lineage tracing and in situ hybridization show that Pax3-Cre derivatives contribute to S100+ , Kir4.1+ and Dct+ melanocytes (intermediate cells) in the developing stria vascularis, all significantly diminished in Pax3 mutant animals. Taken together, these results suggest that Pax3 is required for the development of neural crest cell-derived cochlear melanocytes, whose absence may contribute to congenital hearing loss of Waardenburg syndrome in human.

## Introduction

*Pax3* regulates induction and differentiation from neural crest cells which are critical in the development of many organs such as eye, hair, skin, neural tube, cranium, face and inner ear [1–4]. During embryonic development, *Pax3-Cre*$^{+}$ neuroepithelial cells including neural crest cells migrate into the otic vesicle, which is primordium of the inner ear, and give rise to melanocytes and glial cells in the developing cochlea [5–8].

Human *PAX3* gene mutations cause Waardenburg syndrome which is characterized by telecanthus (widely spaced eyes), heterochromia iridis, patchy pigmentation of hair and skin, and profound sensorineural hearing loss [9–11]. It has been postulated that congenital hearing loss in Waardenburg syndrome results from developmental defects of cochlear melanocytes, which are located as intermediate cells in the stria vascularis and necessary for generating the endocochlear potential required for auditory function [6,12–15]. Although radiographic imaging of temporal bone of patients with *PAX3* mutations show normal inner ear structure, one mouse model of Waardenburg syndrome ($Sp^{2H}$/ $Sp^{2H}$) has been reported to display a complete absence of cochlear components such as the organ of Corti or stria vascularis at late embryonic stages [16,17].

Pax3 deficiency has been postulated to prevent development of *Pax3-Cre*$^{+}$ derivatives including intermediate cells in the developing stria vascularis [6]. Recently, fate-mapping experiments showed that intermediate cells have dual embryonic origins: melanoblasts and Schwann cell precursors, which are derived from neural crest cells and begin to migrate into the stria vascularis around embryonic day (E) 15.5 first in the base and proceeds towards the apex [8].

In this study, we examined *Pax3*$^{Cre/Cre}$ mice which models human Waardenburg syndrome with complete loss of Pax3 protein [18] and found that loss of *Pax3* prevents formation of melanocytes (intermediate cells) in the developing cochlea. At E18.5, *Pax3* knockout cochleae showed 2 major phenotypes: 57% animals showed smaller cochleae with normal organization of the organ of Corti, whereas 43% showed severely shortened cochlea in addition to gross organ dysgenesis such as exencephaly. Furthermore, with fatemapping and immunostaining for cochlear melanocytes, we uncovered that S100$^+$ Pax3$^{Cre}$-EGFP$^+$ intermediate cells were diminished in the stria vascularis of E18.5 Pax3 KO cochleae, with remaining those intermediate cells continuing to fully differentiate into *Dct*$^+$ or *Kir4.1*$^+$ melanocytes. Our results suggest that loss of *Pax3* leads to loss of cochlear melanocytes, which may cause congenital hearing loss of Waardenburg syndrome.

## Results

#### Auditory function and cytoarchitecture of the Pax3 $^{Cre/+}$ mice

To determine the cell fate of *Pax3-Cre* derivatives in the cochlea, we used a lineage tracing approach (*Pax3-Cre; CAG-CAT-EGFP or Rosa-mTmG*) where *Pax3-Cre* derived cells are labeled with EGFP. This approach has been used previously to fate-map derivatives of *Pax3-Cre*$^+$ neuroepithelial cells including neural crest cells in various organs including the cochlea [5,6,18,19]. We first confirmed that Pax3$^{Cre}$-EGFP$^+$ cells were distributed primarily in the stria vascularis and modiolar regions, while occasionally were detected in the organ of Corti and greater epithelial ridge (GER) region in the E18.5 and postnatal day 1 (P1) *Pax3*$^{Cre/+}$ heterozygous cochleae (Fig. 1A-B). We also confirmed that *Pax3*$^{Cre/+}$ heterozygous embryos had normal cochlear development (Fig. 1A-B), consistent with the previous reports [5,6]. We further assessed auditory responses of adult P56 *Pax3*$^{Cre/+}$ heterozygous mice and found that they had auditory brain responses comparable to wildtype littermates at all frequencies tested (8, 16, 32 kHz) (Fig. 1C).

Like wildtype control cochlea, the P43 *Pax3*$^{Cre/+}$ heterozygous cochlea displayed normal cytoarchitecture of the stria vascularis, organ of Corti and spiral ganglion (Fig. 1D-E). These data indicate that *Pax3*$^{Cre/+}$ heterozygous cochlea serves as a normal control for *Pax3*$^{Cre/Cre}$ homozygous cochlea and also as an excellent model to examine the *Pax3-Cre* derived cellular populations in the cochlea.

#### Pax3 knockout embryos show cochlear and vestibular defects in the late embryonic period

During normal development, the otic vesicle rapidly expands and gives rise to the cochlear duct and vestibular apparatus shortly after E10.5 [20–22]. *Pax3*$^{Cre/Cre}$ homozygous embryos display several developmental anomalies including loop tail and spina bifida with complete loss of Pax3 protein [18]. The degrees of anomaly are classified as mild or severe, with the latter showing exencephaly and shortened cochlea at E12.5 and E14.5, and then *Pax3*$^{Cre/Cre}$ homozygous embryos also die by P0 [5,6,18]. Thus, we characterized the morphology of embryos shortly before birth at E18.5 (27 wildtype, 37 *Pax3*$^{Cre/+}$ heterozygous, 14 *Pax3*$^{Cre/Cre}$ homozygous embryos from 7 litters). We found that 8 *Pax3*$^{Cre/Cre}$

homozygous embryos displayed mild anomalies (mostly loop tail and spina bifida) (**Supplementary Fig. S1A-C, Table S1**), and 6 were severe (visibly smaller, displayed exencephaly, loop tail, and spina bifida) (**Supplementary Fig. S1D, Table S1**).

To further examine the morphology of the inner ear of *Pax3*$^{Cre/Cre}$ homozygous mice, we performed paint-fill of cochleae from E15.5 *Pax3*$^{Cre/Cre}$ homozygous embryos using wildtype as controls. The inner ear of E15.5 *Pax3*$^{Cre/Cre}$ homozygous mice with a mild phenotype was smaller but exhibited similar morphology to wildtype control (Fig. 2A-B). However, *Pax3*$^{Cre/Cre}$ homozygous embryo with a severe phenotype showed several malformations of both vestibular and cochlear organs, including underdeveloped semicircular canals, vestigial endolymphatic duct, and foreshortened cochlear duct (Fig. 2C). Similarly, at E18.5, *Pax3*$^{Cre/Cre}$ homozygous inner ear with severe phenotype was noticeably smaller than wildtype and *Pax3*$^{Cre/+}$ heterozygous (Fig. 2D-G). These results indicate that development of the inner ear is grossly normal in *Pax3*$^{Cre/Cre}$ homozygous embryo with mild phenotype but is severely perturbed in *Pax3*$^{Cre/Cre}$ homozygous embryo with severe phenotype.

**Some Pax3-Cre derivatives distribute as the intermediate cells in the stria vascularis of Pax3 knockout cochleae**

Lineage tracing experiments using *Wnt1-Cre*, *Plp1-Cre* and *Pax3-Cre* mice demonstrate that neural crest cells migrate and develop as glial cells including Schwann cells and satellite cells in the spiral ganglion and the cochlear melanocytes (intermediate cells) in the stria vascularis [5,6,8].

We first determined the cellular contribution of the *Pax3-Cre* lineage in the spiral ganglion region. At E18.5, we found Pax3$^{Cre}$-EGFP$^{+}$ cells populating the spiral ganglion regions (Fig. 1A, 3A), consistent with the previous reports [5,6]. At E18.5, *Pax3*$^{Cre/Cre}$ homozygous cochleae appeared smaller than *Pax3*$^{Cre/+}$ heterozygous cochleae, although Pax3$^{Cre}$-EGFP$^{+}$ cells remained present in the spiral ganglion regions throughout the cochlea in the both mild and severe homozygous animals (Fig. 1A, 3A-B, **Supplementary Fig. S2A-B**).

The mature stria vascularis has three cell types: marginal cells, basal cells and intermediate cells, the latter of which are melanocytes. By contrast, the embryonic stria vascularis is composed of only marginal cells and intermediate cells [23]. Previously, *Pax3*$^{Cre/Cre}$ homozygous cochlea has been shown to display a complete loss of *Pax3-Cre* derivatives and *Dct*$^{+}$ cochlear melanocytes in the stria vascularis at E15.5 [6]. As melanocytes originate from both melanoblasts and Schwann cell precursors [8], we hypothesize that Pax3 deficiency leads to a partial, and not complete, loss of cochlear melanocytes at the late embryonic period. First, we analyzed the distribution of the *Pax3-Cre* derivatives in the stria vascularis of the E18.5 *Pax3*$^{Cre/Cre}$ homozygous cochlea. Kcnq1 is a marker for the marginal cells, and S100 marks both the marginal cell and intermediate cells in the stria vascularis [23,24]. In the *Pax3*$^{Cre/+}$ heterozygous cochlea, many S100$^{+}$ Pax3$^{Cre}$-EGFP$^{+}$ intermediate cells were detected next to Kcnq1$^{+}$ S100$^{+}$ marginal cells in the stria vascularis of all turns (Fig. 3A). By contrast, in the E18.5 *Pax3*$^{Cre/Cre}$ homozygous cochlea (mild phenotype), few or no S100$^+$ Pax3$^{Cre}$-EGFP$^+$ intermediate cells were detected in the stria vascularis of the apical (Fig. 3B‴) and middle turns (Fig. 3B′, **B⁗**), while some S100$^+$ Pax3$^{Cre}$-EGFP$^+$ intermediate cells appeared in the basal turn (Fig. 3B⁗).

Next, we quantified intermediate cells and compared the *Pax3*$^{Cre/Cre}$ homozygous (mild phenotype) cochlea with that of the *Pax3*$^{Cre/+}$ heterozygous or wildtype cochlea at E18.5. All of them displayed four cochlear regions (apex, mid-apex, mid-base and base) in cross-section (Fig. 1A, 3A-B, 4A-B, 5A-B, **Supplementary Fig. S2A**). In each cochlear turn, there were noticeably fewer S100$^+$ Pax3$^{Cre}$-EGFP$^+$ intermediate cells in the stria vascularis of *Pax3*$^{Cre/Cre}$ homozygous embryos with mild phenotype than *Pax3*$^{Cre/+}$ heterozygous embryos. The reduction is most dramatic in the apical turn relative to the base (Fig. 3C). These data suggest that loss of *Pax*3 prevents normal development of intermediate cells in the developing cochlea, with the apical and middle cochlear turns more severely affected than the base.

**Characterizing melanocytes in the stria vascularis in the Pax3 knockout embryos**

To further characterize whether cochlear melanocytes were perturbed in *Pax3*$^{Cre/Cre}$ homozygous mouse, we performed *in situ* hybridization for markers of melanocytes. *Dct*, a classical marker of melanocytes, was detected in all three turns in the E18.5 wildtype cochlea (Fig. 4A). In the *Pax3*$^{Cre/Cre}$ homozygous cochlea (mild phenotype), we discovered markedly fewer *Dct*$^+$ melanocytes in all turns, with the greatest reduction observed in the apical turn (Fig. 4B, C).

Moreover, we examined expression of the inwardly rectifying potassium channel *Kir4.1*, whose expression in the stria vascularis is crucial for development of the endocochlear potential after P7 [12,13,25]. Because *Pax3*$^{Cre/Cre}$ homozygous mice are lethal perinatally, we investigated *Kir4.1* mRNA expressions in the E18.5 cochleae [18]. *Kir4.1* mRNA expression was detected in the stria vascularis of both control and the *Pax3*$^{Cre/Cre}$ homozygous mild phenotype cochlea. *Kir4.1* mRNA expression was also detected in the organ of Corti and spiral ganglion (Fig. 5A-B). In the *Pax3*$^{Cre/Cre}$ homozygous embryos with mild phenotype, there were significantly fewer *Kir4.1*$^+$ cells in the stria vascularis in the mid-basal and basal turns than those in *Pax3*$^{Cre/+}$ heterozygous embryos, although the apical and mid-apical turns showed no significant reduction in the number of *Kir4.1*$^+$ cells between those groups (Fig. 5C). Together, these data reveal that Pax3 deficiency perturbs development of cochlear melanocytes.

## Discussion

Waardenburg syndrome is characterized by hearing loss and developmental abnormalities of melanocytes [11,26]. Genetic studies suggest that Waardenburg syndrome is caused by mutations of *PAX3* and other genes such as *MITF*, *SOX10*, *EDN3*, *EDNRB* and *SNAI2*[1,9,27–30]. Here, we used a mouse model of Pax3 deficiency to study Waardenburg syndrome and found that loss of *Pax3* causes a reduction of melanocytes in the developing cochlea, possibly stemming from a disruption to the distribution of neuroepithelial cells including neural crest cells. We showed that *Pax3*$^{Cre/Cre}$ homozygous cochlea was short and vestibular system was malformed. Moreover, we revealed that loss of *Pax3* decreased the number of melanocytes (intermediate cells) in the stria vascularis.

Sensory epithelial cells in the inner ear are mostly derived from the otic vesicle [5,22]. Neuroepithelial cells including neural crest cells have also been proposed to contribute to the otic vesicle and later the sensory epithelium [5]. Neural crest cells detached from the neural tube ectoderm migrate to the otic vesicle and differentiate into various cell types such as melanocytes, Schwann cells and satellite cells in the developing cochlea [31]. Humans carrying *PAX3* mutations have abnormal development of melanocytes, manifested as heterochromia [1]. They also present with profound hearing loss despite grossly normal inner ear structures with radiographic studies [17]. Here we show that loss of *Pax3* causes shortened cochlea and malformed vestibular apparatus, while having cochlear structures such as the stria vascularis. Our study stands in contrast to the results of previous work using $Sp^{2H}/Sp^{2H}$ homozygous embryo, in which deletion of *Pax3* prevented the formation of stria vascularis in the late embryonic cochlea [16]. This difference may be due to a dosage effect of different *Pax3* mutations. Furthermore, $Pax3^{Cre/Cre}$ homozygous embryos in our study represent two distinct severities of phenotype. Those diversities in phenotype may be also related to variable degrees of hearing loss in Waardenburg syndrome caused by various *PAX3* gene mutations [32–34].

Neural crest cells with multi-potent potentials differentiate into the various cell types including melanocytes and play important roles in the development of various organs [2,18]. Melanocytes originating from neural crest cells migrate into specific locations within the skin and hair follicles, and to other sites including stria vascularis in the cochlea [35]. Cochlear melanocytes are known as intermediate cells which generate high concentration of potassium ion in the cochlear endolymph [13,36]. Endocochlear potential in-turn drives depolarization of hair cells and is required for hearing function [37]. It has been thought that the developmental disorder of cochlear melanocytes causes Waardenburg syndrome [6,9,27,38,39]. In this study, we demonstrated with a model of Waardenburg syndrome that *Pax3* is necessary for the correct distribution of cochlear melanocytes, whilst an intermediate cell marker, S100, expressing *Pax3-Cre* derivatives from neuroepithelial cells distributed and $Dct^{+}$ or $Kir4.1^{+}$ cochlear melanocytes were localized as intermediate cells in the stria vascularis at late embryonic day. Furthermore, our study pointed out that small number of cochlear melanocytes can still development despite Pax3 deficiency, suggesting that they do not always require Pax3 and may develop with any redundant pathway. In normal development, it is newly reported that cochlear melanocytes develop from Schwann cell precursors derived from neural crest cells, starting to migrate into the stria vascularis around middle embryonic day (E15.5) [8]. Interestingly, a previous report found no *Pax3-Cre* derivatives or $Dct^{+}$ melanocytes in the stria vascularis at E15.5, although the distribution of *Pax3-Cre* derivatives remained normal in the glial cell region [6]. In contrast, we observed *Pax3-Cre* derivatives in both the glial cell region and stria vascularis, and $Dct^{+}$ or $Kir4.1^{+}$ melanocytes in the stria vascularis in the late embryonic period (E18.5). These divergent findings may be indicative of a delayed migration of *Pax3-Cre* derived cells from the glial region to other domains within the cochlea in this model of Waardenburg syndrome.

Finally, our data exhibited a degree of variance in the number of cochlear melanocytes in the stria vascularis of Waardenburg syndrome model caused by loss of *Pax3*. This finding may be reflective in the degree of hearing loss from in Waardenburg syndrome [32–34]. The developing melanocytes in human cochlea are considered as the prime target cells of gene therapy for Waardenburg syndrome [30]. Our results indicate that neuroepithelial cells in a Waardenburg syndrome model could differentiate as melanocytes if they properly migrate into the stria vascularis. In conclusion, our data would guide future studies to develop hearing therapies for Waardenburg syndrome.

## Methods

**Mice**: The following mouse strains were used: $Pax3^{Cre/+}$ (Stock #005549, Jackson Laboratory) [18], $CAG^{CAT-EGFP/+}$ (gift from J. Miyazaki, Osaka Univ.) [40], $R26R^{mTmG}$ mice (stock #007576, Jackson Laboratory) [41]. Mouse embryos of both genders were used. Institutional Animal Care and Use Committee of The Jikei University School of Medicine (protocol number: 21–025, 2020-060) and Stanford University School of Medicine (protocol number: 18606) approved all procedures. All experimental procedures were performed in accordance with relevant guidelines and regulations. This study is reported in accordance with ARRIVE guidelines, https://arriveguidelines.org.

### Genotyping

Mouse genomic DNA was isolated from collected tail tips by adding 180 µl of 50 mM NaOH and incubating at 98°C for 10 min, followed by the addition of 20 µl of 1 M Tris-HCl. PCR was performed to genotype transgenic mice with three specific primers which sequences were described in a previous paper [18].

### Auditory physiology measurements

Auditory brainstem responses were recorded as described in a previous paper [42]. Briefly, P56 mice were anesthetized with a ketamine/xylazine mixture (100 mg/kg ketamine and 10 mg/kg xylazine, IP) and placed on a heating pad at 37°C. Auditory brain responses were measured with a needle electrode which was located inferior to the tympanic bulla, referenced to an electrode on the vertex of the head, and a ground electrode was inserted at the hind limb. Tone burst stimuli were delivered with frequencies ranging from 8 to 32 kHz (8.0, 16.0, 32.0 kHz) up to 90 dB sound pressure level (SPL) in 5 dB steps. At each frequency and SPL, 512 trials were tested and averaged.

### H&E staining

P43 mice were deeply anesthetized with pentobarbital sodium (50 mg/kg, IP), and perfused transcardially with 4% paraformaldehyde (PFA) in 0.1 M phosphate-buffered saline (PBS), pH 7.4. Temporal bones were harvested and additionally fixed in 4% PFA overnight at 4°C. After PBS wash, inner ears were dissected from temporal bones and decalcified in 0.125 M EDTA for 2 weeks. Inner ears were dehydrated, embedded in paraffin wax, and sectioned to 4 µm using microtome REM-710 (YAMATO). Then, sections were stained with Hematoxylin and Eosin.

**Immunohistochemistry**: Methods were modified as previously reported [43,44]. Briefly, E18.5 heads were harvested and fixed in 4% PFA overnight at 4°C and then embedded in Tissue Tek OCT compound (Sakura, Tokyo, Japan) and frozen. Sections (10 µm thickness) were prepared using a cryostat CM3050S (Leica). P1 whole mount cochleae were dissected and fixed in 4% PFA for 1 hour at 4°C room temperature (RT). Tissues were permeabilized with 0.5% TritonX-100 in PBS for 1 hour at RT, and then blocked with 10% goat or donkey serum, 0.1% TritonX-100 and 1% bovine serum albumin in PBS for 30 min at RT. The following primary antibodies were used: chicken anti-GFP (1:1,000, GFP-1020, Aves labs), rabbit anti-Myosin7a (1:1,000; Proteus Bioscience), goat anti-Kcnq1 (1:100, sc-10646, Santa Cruz Biotechnology) and rabbit anti-S100 (ready-to-use liquid, GA504, Dako). Primary antibodies were applied overnight in a humidified chamber at 4°C. The following day, tissues were washed with PBS three times at 5 min intervals and then incubated with Alexa Fluor 488 secondary antibodies (488, 546 or 647,1:500, Invitrogen or Jackson ImmunoResearch) which were diluted in PBS containing 0.1% TritonX-100 and 1% bovine serum albumin for 2 hours at RT. DAPI (1:10,000, Invitrogen) was also used for nuclear staining.

**In situ hybridization**: Harvested E18.5 heads were fixed in 4% PFA overnight at 4°C, embedded for cryosections and sliced into 10 µm sections as described above. RNA probe synthesis and section *in situ* hybridization were performed as previously described with some modifications [44]. Briefly, a digoxigenin-labeled antisense RNA probe was synthesized using the DIG RNA Labeling Kit (Promega) with plasmids containing the following mouse genes: *Dct* (forward primer, TCCCGAGGCAACCAACATCT; reverse primer, CAGTAGGGCAACGCAAAGGA) and *Kir4.1* (forward primer, GGACAAACCCTTATCTGATTCCA; reverse primer, TGCGCAATAAGAAGCACGAT). Slides were permeabilized with protein K (Roche, 5 µg/ml) in PBS with 0.1% Tween 20 for 10 min at 37°C, incubated with 1 µg/ml digoxigenin-labeled riboprobe in hybridization buffer for 16 hours at 70°C, blocked with 10% heat-inactivated sheep serum in Tris-buffered saline containing 0.1% Tween 20 for 30 min at RT, incubated with anti-digoxigenin antibody-conjugated alkaline phosphatase (1:2000, Roche) in Tris-buffered saline containing 0.1% Tween 20 and 1% heat-inactivated sheep serum for 2 hours at RT. Antibody detection was performed by incubating slides with 0.2% nitroblue tetrazolium and 0.2% 5-Bromo-4-chloro-3-indolyl phosphate p-toluidine salt in detection solution (0.1 M NaCl, 0.1 M Tris-HCl [pH 9.5], 50 mM MgCl2, 1% Tween 20) for 40–48 hours at RT.

### Imaging and cell quantification

Section cochleae were captured using Axio Imager D1 (Zeiss) for bright field images or LSM500/880 (Zeiss) for fluorescent images. Image analyses were performed using Zen Software (Zeiss) and Photoshop CS6 (Adobe Systems). Cells were quantified from each turn (apical, mid-apical, mid-basal and basal turn) in the developing stria vascularis of section images. Melanocytes in the stria vascularis were identified as *Dct*$^+$ or *Kir4.1*$^+$ cells immediately next to marginal cells.

### Statistical analyses

Statistical analyses were performed using Microsoft Excel (Microsoft) and GraphPad Prism 7.03 (GraphPad). Two-way analysis of variance (ANOVA) was used for comparison with two independent variables. $P < 0.05$ was considered statistically significant.

#### Paint injection

Paint-filling of the inner ear was performed as previously described [22]. E15.5 mouse embryos were harvested and fixed overnight in Bodian's fixative. Samples were then dehydrated with ethanol and cleared with methyl salicylate. Glass micropipette was inserted in the utricle, and then inner ears were visualized by injecting white latex paint in 0.1% methyl salicylate into the membranous labyrinth. Samples were captured with stereomicroscope SZ61 (Olympus).

#### Morphological picture

Harvested E18.5 mouse embryos were captured and then temporal bones were harvested and fixed in 4% PFA overnight at 4°C. Following several PBS washes, inner ears were isolated from temporal bones including cochlear capsule in PBS. Samples were captured with stereomicroscope Lumar.V12 (Zeiss).

### Declarations

Author Contributions: T.U., E.T., N.T., H.M. designed experiments, T.U., E.T., N.T., H.M. performed experiments, T.U., E.T., N.T., H.M., Y.K. analyzed data, T.U., E.T., N.T., H.M., T.M., P.J.A., M.Y., H.K., M.O., A.G.C wrote the manuscript.

Competing interests: The authors declare no competing interests.

#### Data availability

All data analyzed in this study are included in this article and its supplementary Information files. Mouse nucleotide sequences of *Dct* (accession number: NM_010024.3) and *Kir4.1* (accession number: NM_001039484.1) genes were referenced from the National Institutes of Health (NIH) genetic sequence database GenBank, https://www.ncbi.nlm.nih.gov/genbank/.

**Acknowledgements:** We thank our laboratory for insightful comments on the manuscript, J. Miyazaki (Osaka Univ.) for mouse sharing, and K. Yokota and E. Hayashi (Jikei Univ.) for excellent technical support. This work was supported by a Grant-in-Aid for Young Scientists from the Ministry of Education, Culture, Sports, Science and Technology, Japan, KAKENHI (25861596) (T.U.), (20K22986) (E.T.), (20890228) (N.T) and NIDCD/NIH RO1DC021110 (A.G.C.).

## Figures

Figure 1

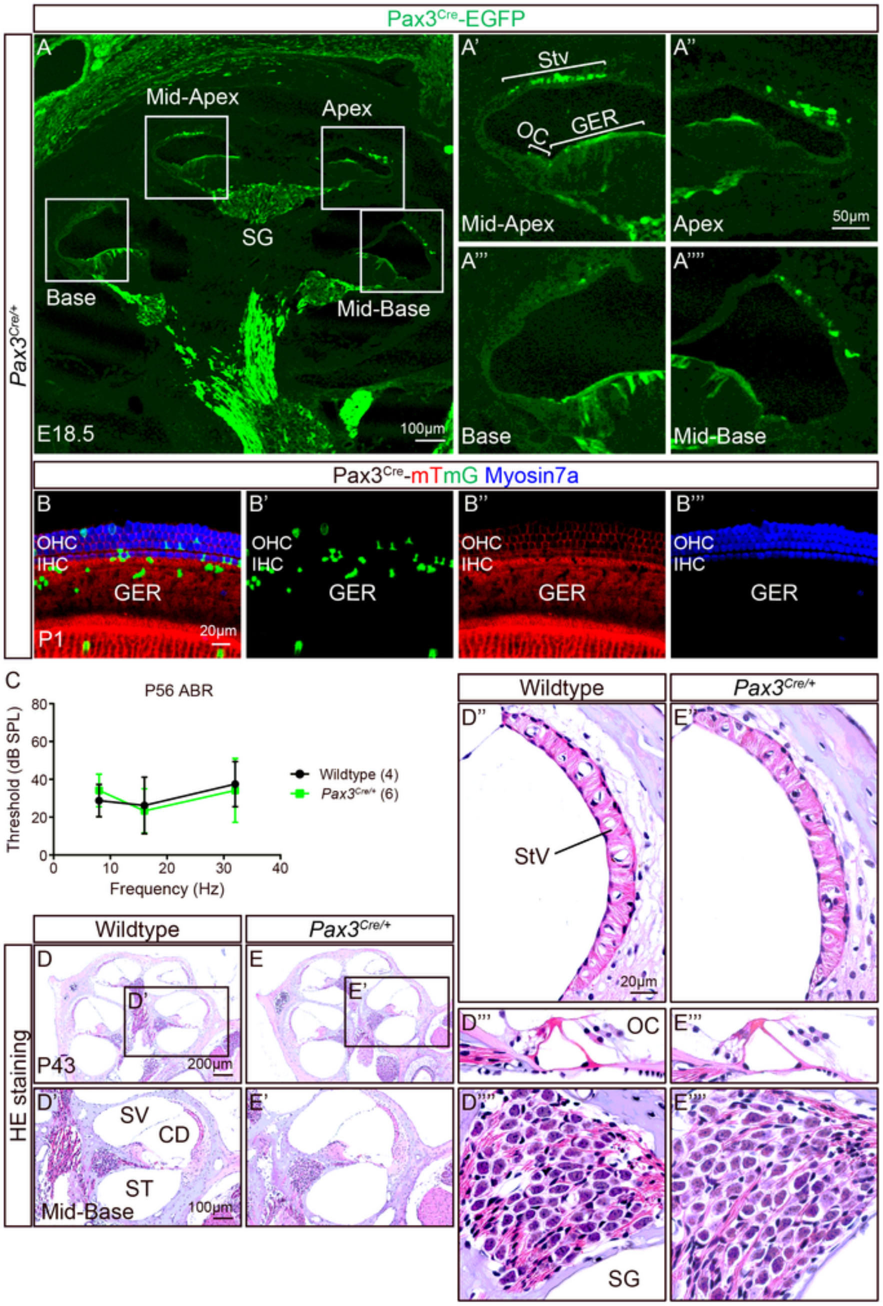

### Figure 1

**Auditory and cochlear properties of *Pax3$^{Cre/+}$* heterozygous mice.** (A-A'''') In the E18.5 *Pax3$^{Cre/+}$* cochlea, Pax3$^{Cre}$-EGFP$^{+}$ cells distributed in the stria vascularis and glial cell region including spiral ganglion, GER and organ of Corti. (B) Some Pax3$^{Cre}$-EGFP$^{+}$ cells scattered in the GER and organ of Corti at P1. (C) P56 *Pax3$^{Cre/+}$* heterozygous mice had no significant difference in auditory brain responses thresholds at all frequencies compared with wildtype. (D-E) P43 cochlea showed no difference in the stria vascularis, organ of Corti or spiral ganglion neuron between wildtype and *Pax3*$^{Cre/+}$ heterozygous mouse. ABR, auditory brain responses; CD, cochlear duct; GER, greater epithelial ridge; IHC, inner hair cell; SV, scala vestibuli; ST, scala tympani; StV, stria vascularis; OC, organ of Corti; OHC, outer hair cell; SG, spiral ganglion; data represent mean±S.D. (two-way ANOVA with Sidak's multiple comparisons test). n=4-6.

Figure 2

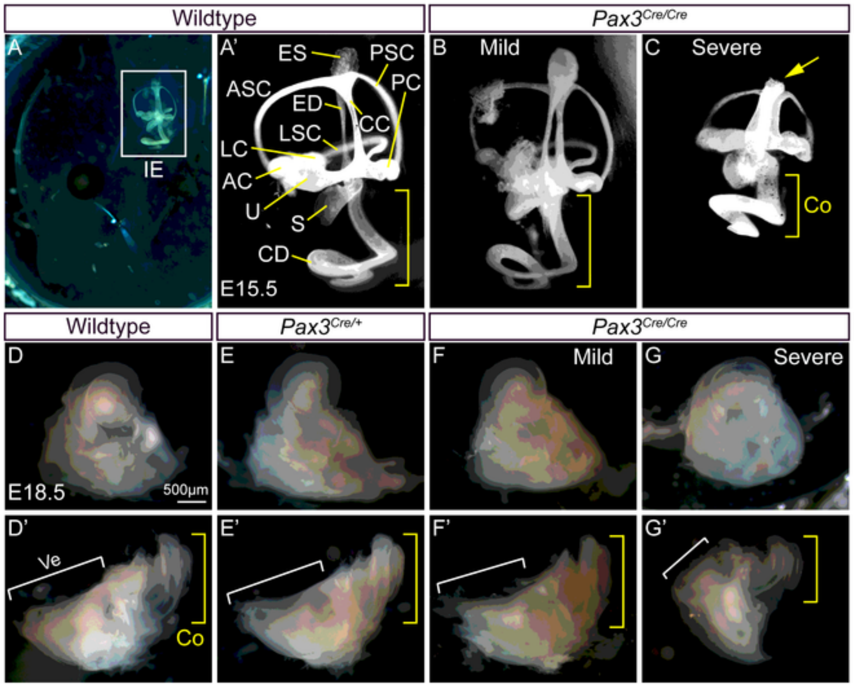

### Figure 2

***Pax3 knockout* inner ear phenotypes.** (A-A’) White latex paint was injected into the endolymph duct in the E15.5 wildtype mouse embryo. (B-C) Two pattern shapes (mild and severe) of the endolymph duct in the E15.5 *Pax3 knockout* cochlea. Compared with two types of *Pax3* knockout inner ear, severe phenotype displayed shortened cochlear duct (yellow bracket) and semicircular canals, and diminished endolymphatic sac (yellow arrow) than mild phenotype. (D-G) Compared with other E18.5 samples, severe phenotype of the E18.5 $Pax3^{Cre/Cre}$ homozygous inner ear showed smaller cochlear (yellow bracket) and vestibular organs (white bracket). IE, inner ear; CD, cochlear duct; U, utricle; S, saccule; AC, anterior crista; PC, posterior crista; LC, lateral crista; ASC, anterior semicircular canal; PSC, posterior semicircular canal; LSC, lateral semicircular canal; CC, common crus; ED, endolymphatic duct; ES, endolymphatic sac; D, dorsal; P, posterior; Co, cochlear organ; Ve, vestibular organs.

Figure 3

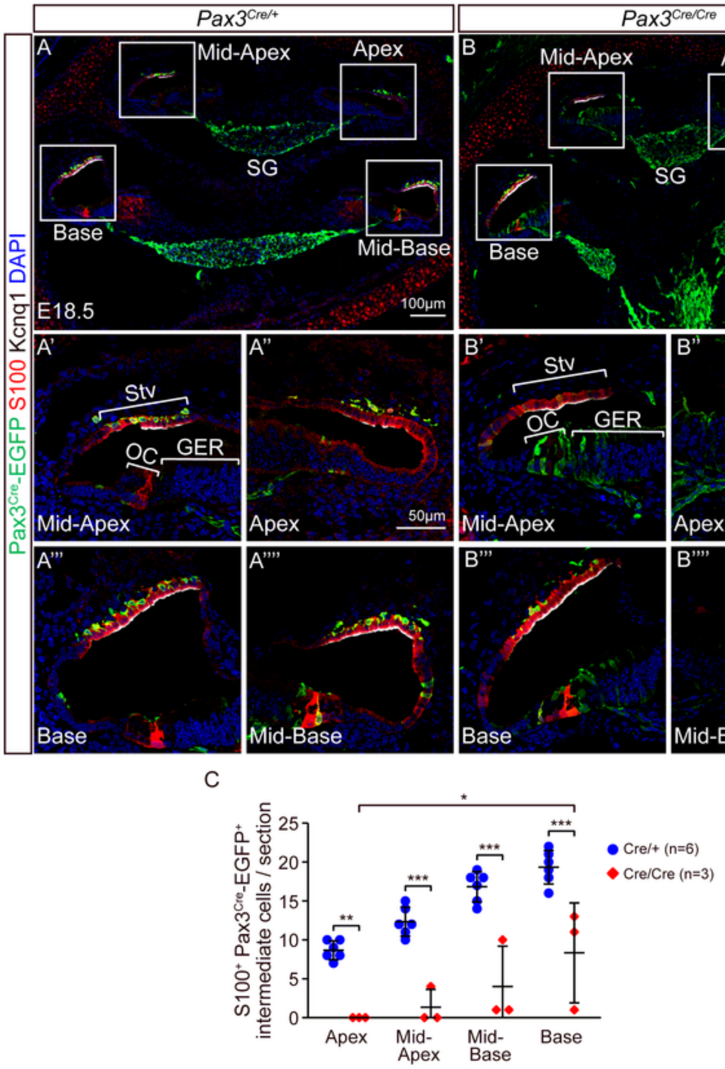

### Figure 3

**Fewer intermediate cells derived from *Pax3-Cre* derivatives in the *Pax3 knockout* cochleae.** (A-A'''') In the E18.5 $Pax3^{Cre/+}$ control cochlea, $S100^+$ $Pax3^{Cre}$-$EGFP^+$ intermediate cells were localized besides $Kcnq1^+$ $S100^+$ marginal cells in the stria vascularis of all cochlear turns. (B-B'''') In the stria vascularis of the E18.5 $Pax3^{Cre/Cre}$ mild phenotype cochlea, some $S100^+$ $Pax3^{Cre}$-$EGFP^+$ intermediate cells were detected next to

Kcnq1$^+$ S100$^+$ marginal cells. (C) Quantification of S100$^+$ Pax3$^{Cre}$-EGFP$^+$ intermediate cells in the stria vascularis showed significant reductions of all turns in the E18.5 *Pax3$^{Cre/Cre}$* mild phenotype homozygous embryos compared with *Pax3$^{Cre/+}$* heterozygous embryos. The basal turn contained significantly more S100$^+$ Pax3$^{Cre}$-EGFP$^+$ intermediate cells than the apical turn in the stria vascularis of the E18.5 *Pax3$^{Cre/Cre}$* mild phenotype homozygous embryos. StV, stria vascularis; OC, organ of Corti; SG, spiral ganglion; GER, greater epithelial ridge; data represent mean±S.D. *$p<0.05$ **$p<0.01$ ***$p<0.001$ (two-way ANOVA with Tukey's multiple comparisons test). n=3-6.

Figure 4

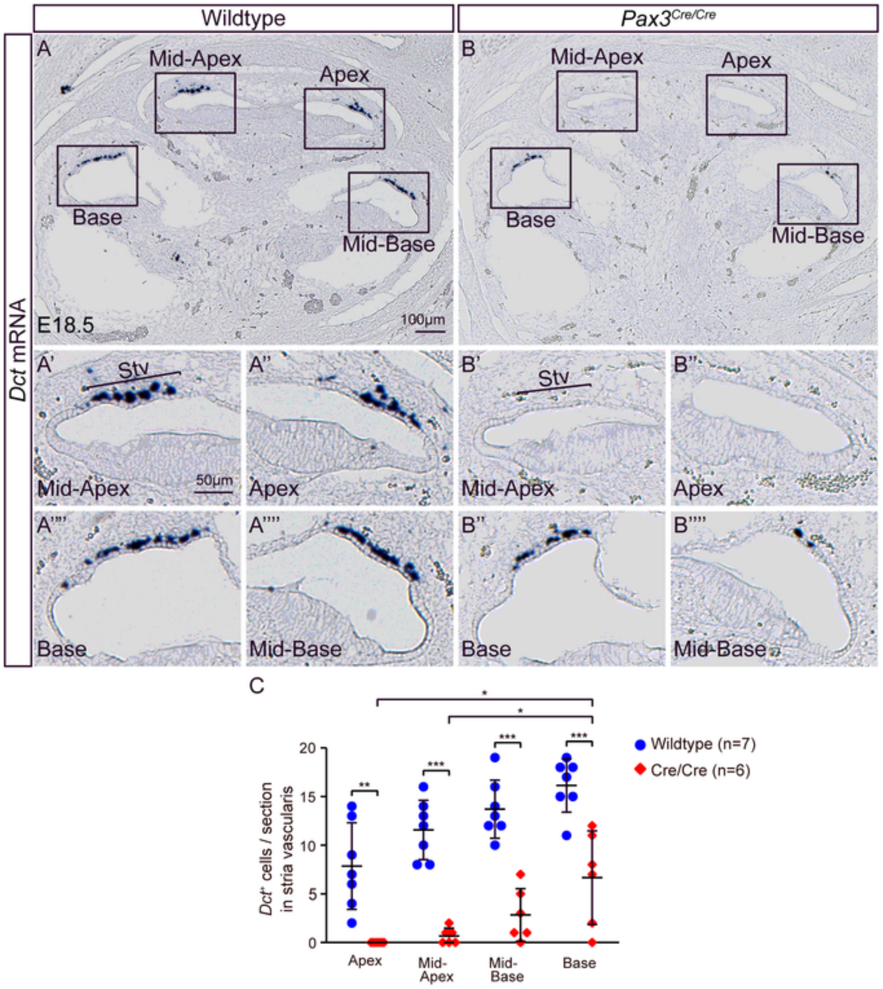

**Figure 4**

**Distribution of melanocytes in the *Pax3 knockout* cochleae at late embryonic day.** (A-A'''') In the E18.5 *Pax3*$^{Cre/+}$ control cochlea, *Dct*$^{+}$ melanocytes were detected on a straight line along with the cochlear duct in the stria vascularis of all cochlear turns. (B-B'''') In the stria vascularis of the E18.5 *Pax3*$^{Cre/Cre}$ mild phenotype cochlea, no *Dct*$^{+}$ melanocytes were found in the apical or mid-apical turn although a few *Dct*$^{+}$

melanocytes were detected in the mid-basal or basal turn. (C) *Dct*$^+$ melanocytes in stria vascularis were significantly fewer in all turns of the E18.5 *Pax3*$^{Cre/Cre}$ mild phenotype homozygous cochleae than E18.5 wildtype cochleae. The basal turn had significantly more *Dct*$^+$ melanocytes than the apical and mid-apical turns in the E18.5 *Pax3*$^{Cre/Cre}$ mild phenotype homozygous embryos. StV, stria vascularis; data represent mean±S.D. *$p<0.05$, **$p<0.01$ ***$p<0.001$ (two-way ANOVA with Tukey's multiple comparisons test). n=6-7.

Figure 5

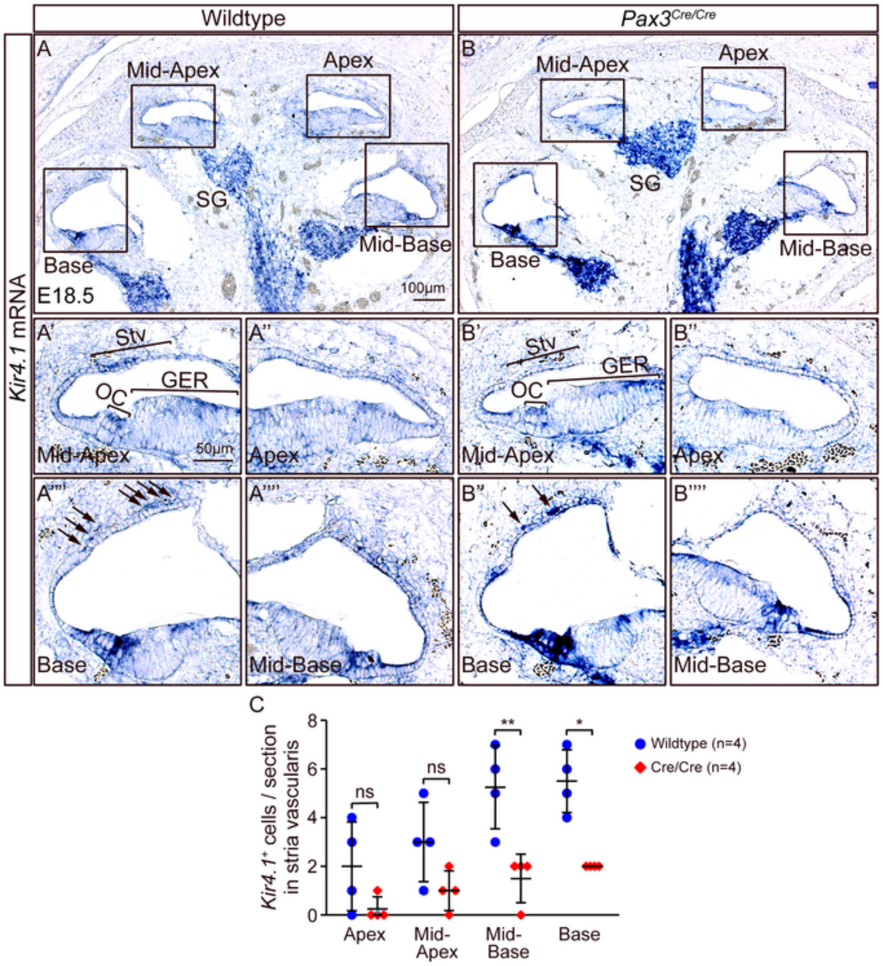

#### Figure 5

***Pax3 knockout*** **cochleae exhibit some differentiated intermediate cells in the stria vascularis in late embryonic stages.** (A-B) A few *Kir4.1*$^{+}$ intermediate cells (arrows) were found in the stria vascularis of each turn of the E18.5 *Pax3*$^{Cre/+}$ control and *Pax3*$^{Cre/Cre}$ mild phenotype cochlea. (C) Quantification showed significant reductions of *Kir4.1*$^{+}$ melanocytes in the stria vascularis of the mid-basal and basal turns in the E18.5 *Pax3*$^{Cre/Cre}$ mild phenotype homozygous embryos compared with the E18.5 *Pax3*$^{Cre/+}$ heterozygous embryos. StV, stria vascularis; OC, organ of Corti; SG, spiral ganglion; GER, greater epithelial ridge; data represent mean±S.D. *$p<0.05$ **$p<0.01$ (two-way ANOVA with Tukey's multiple comparisons test). n=4.

## Supplementary Files

This is a list of supplementary files associated with this preprint. Click to download.

- SupplementaryFigureS1.tif
- SupplementaryFigureS2.tif
- SupplementaryTableS1.xlsx
- SupplementaryItems.docx
